# Structural equation model for parental influence on children’s oral health practice and status

**DOI:** 10.1186/s12903-020-1048-2

**Published:** 2020-02-17

**Authors:** Yan Zhang, Kar Yan Li, Edward Chin Man Lo, May Chun Mei Wong

**Affiliations:** 1 Dental Public Health, Faculty of Dentistry, The University of Hong Kong, Pofulam, Hong Kong, SAR China; 2 Faculty of Dentistry, The University of Hong Kong, Pofulam, Hong Kong, SAR China

**Keywords:** Oral health practice, Oral health status, Children, Parental influence

## Abstract

**Background:**

Children’s oral health status (OHS) can be influenced by their oral health practices and many parental factors. This study aimed to investigate pathways from parental factors to oral health practices and status of children in Hong Kong.

**Methods:**

Using a combination of random and purposive sampling of Hong Kong households, 432 families with children aged 5–7 participated in a cross-sectional survey. Data on socioeconomic status, smoking, and oral health knowledge, attitudes, and practices, as well as OHS of parents and parents’ knowledge of and attitudes towards their children’s oral health, were collected through a questionnaire. Tooth status, periodontal status, and oral hygiene data were also collected through clinical examination. Correlations of oral health behaviors (OHB) and OHS within families were assessed by confirmatory factor analysis. A conceptual model of the parental influences on children’s oral health practices and status was tested by a structural equation model (SEM). Chi-square test, chi-square/df, nonnormed fit index, comparative fit index, and root mean square error of approximation were used to assess the model fit.

**Results:**

Fit indexes for confirmatory factor analysis and SEM showed good fit. Positive correlations of OHB and OHS were found within the families that ranged from 0.74 to 0.98 for OHB and 0.30 to 0.43 for OHS. SEM showed better socioeconomic status of mothers led to better oral health knowledge and attitude (γ = 0.75, *P* < 0.001) and also towards their children’s better oral health knowledge and attitude (γ = 0.44, *P* < 0.01). Parents’ attitudes towards their children’s oral health (β = 0.40, *P* = 0.04) and mothers’ OHB (β = 0.60, *P* < 0.001) were positively associated with OHB of children. Positive OHB of children (β = − 0.48, *P* < 0.01) in turn led to better oral health.

**Conclusions:**

Correlations of OHB and OHS between mothers and children were stronger than those of fathers. Children’s OHS was directly affected by their mothers’ OHB, which in turn were affected by parents’ oral health knowledge, attitudes, and practices.

## Background

Children’s oral health status (OHS) can be influenced by their oral health practices and many parental factors [1]. Children’s oral health practices and statuses were found to be associated with their parents’ income and education levels [2, 3]. Several studies found that children from families with higher incomes experienced fewer dental caries [4–6]. Moreover, a higher education level was related to a higher income, thus a better occupation and more opportunities to receive health education. Higher education levels of mothers were also related to better oral hygiene status of their children [7]. A study of Hong Kong preschool children found that mothers’ and fathers’ education levels were negatively related to children’s caries experience [8]. A cross-sectional study of 12-year-old Libyan children showed that the caries experiences of children as measured by DMFT scores were negatively associated with their fathers’ education levels [9]. Other studies found that low education levels of parents were associated with their children’s dental caries incidence in primary dentition and permanent dentition [10–13].

In a family, parents may affect their children’s oral health behaviors (OHB) and OHS via their oral health knowledge, attitudes, and behaviors. A study on low-income Africa American preschool children found that the mothers’ knowledge of their children’s oral hygiene was associated with their children’s tooth-brushing frequency [14]. With an increase in one unit of knowledge score, the children’s tooth-brushing frequencies would be increased by 22 and 13% in the 1–3 and 4–5 age groups, respectively. Parents’ oral health knowledge is not just related to children’s tooth-brushing frequency but also children’s oral hygiene status and dental caries experience. It was reported that parents with better oral health knowledge had a higher chance of having children with better oral hygiene and lower DMFT scores [15]. Children whose mothers demonstrated less knowledge of oral hygiene were prone to having early childhood caries [14].

Parents’ oral health attitudes are related to their oral health knowledge and OHBs. Besides parents’ oral health knowledge, their attitudes can also affect their children’s oral health. A study of children aged 3 to 4 years old and their mothers in 17 countries reported significant differences in parents’ attitudes between those children with and without dental caries [16]. A survey of children 3 to 5 years old reported that both parents’ attitudes to diet and oral hygiene were the risk indicators of dental caries in their children [17]. A cross-sectional study of more than 400 pairs of mothers and preschool children in Nigeria tried to identify the maternal-related risk factors to their children’s dental health status [18]. It found that better oral health attitude of mothers was associated with the absence of dental caries and better oral hygiene of the children.

Health practices of the parents and their children are found to be correlated. Parents functioning as a social model for their children is expected in several dental health practices. Social learning theory is a comprehensive theory used to interoperate the influence of parents’ practices on their children’s practices. Children may emulate and imitate their parents’ practices. OHB are aggregated between parents and their children [19]. Some studies found that mothers’ positive tooth-brushing behavior and use of dental floss were associated with more frequent tooth brushing of their children [14, 20]. A study in Japan also showed that better dental health behavior of parents had a positive effect on their children’s oral health [21].

Some epidemiological studies have examined the parent-child aggregation of OHS and found that parents’ OHS was associated with their children’s OHS. Some researchers reported an association of dental caries experience between parents and children [22, 23]. Self-assessed poor dental health of parents was related to the presence of early dental caries in their children [24]. Self-reported family history of periodontal diseases was also found to be a risk factor of periodontitis [25].

**Fig. 1:**
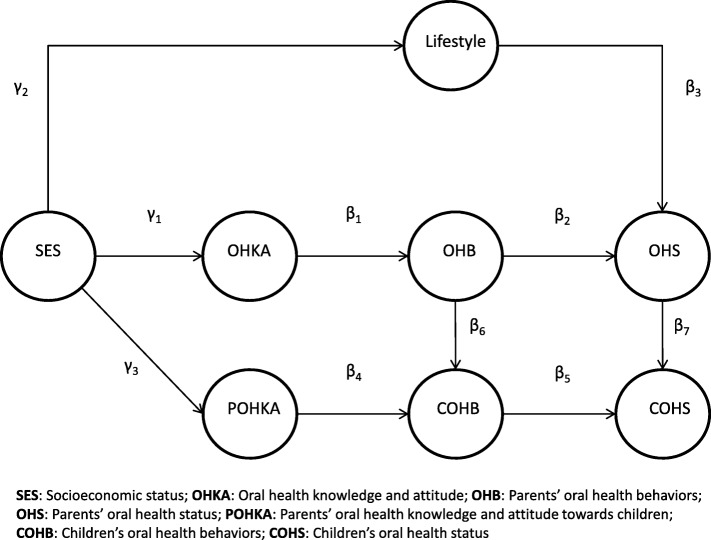
Hypothesized path diagram of Parent-child OHS model

This study aimed to investigate the pathways from parental factors to oral health practice and status of children in Hong Kong. A conceptual model of the pathways from socioeconomic status, oral health knowledge, attitudes, and practices to the OHS of parents based on a previous publication [26] and parental influences on children’s oral health practices and status based on the abovementioned research was hypothesized (Fig. 1) and tested using a structural equation model (SEM). It is hypothesized that the parents’ OHS can be affected by their OHB, oral health knowledge and attitudes, lifestyle and socioeconomic status, whereas the children’s OHS can be affected by these parental factors.

## Methods

### Study participants

This study was a cross-sectional survey using a combination of random household and purposive sampling. The study population, sampling method, and recruitment procedure have been reported previously [26]. In brief, families were recruited from the participants of a local charitable foundation-funded project called the FAMILY project with randomly selected households (https://www.family.org.hk/en/household-survey-2/), as well as families whose children studied at six kindergartens selected through purposive sampling. These kindergartens, as a complement to the participants recruited from the FAMILY project, were located in different districts of Hong Kong to represent the study population. A total of 455 families with children aged 5 to 7 from the FAMILY project baseline database and 105 families from the selected kindergartens were invited to participate in this study. Invitation letters with consent forms were sent to the selected families. The purpose of the study was explained clearly to the participated families and written consent from the parents and for their children was obtained before the data collection. Oral examinations and questionnaire survey were conducted either at participants’ homes or the kindergartens from September 2009 to November 2011.

### Questionnaire

Before undergoing the clinical examination, the fathers and mothers were asked to complete a questionnaire for themselves separately and individually. The questionnaire assessed their oral health knowledge, attitudes, and behaviors, as well as socioeconomic status (Appendix 1). Another questionnaire about their children’s OHB and parents’ knowledge of and attitudes towards their children’s oral health was completed by either one of the parents, usually the mother (Appendix 1).

### Oral examination

The dental examinations were carried out by a trained and calibrated dentist for all of the study participants. An intraoral LED light, disposable front-surface mirror, and Community Periodontal Index (CPI) probe were used. Information on tooth status, periodontal status, and oral hygiene status of the participants were recorded. The DMF/dmf index was employed to assess their tooth status. The CPI and loss of attachment (LoA) were used to assess parents’ periodontal status. The examining methods followed the recommendations of the WHO [27]. For children, the gingival bleeding index (GBI) was used to assess the gingival health status [28]. The presence or absence of bleeding within 10 s after running a probe along the gingival margin of the upper and lower central incisors and the primary second molars was recorded. The oral cleanliness of the participants was assessed and recorded by the visible plaque index (VPI) devised by Ainamo and Bay [28]. The presence or absence of visible dental plaque on the buccal and lingual surfaces of the right upper central incisor, left lower central incisor, and all first molars were examined for adults. For children, the index teeth were the right primary upper central incisor, left primary lower central incisor, and all primary second molars. Calibration of the examiner with the expert dentist before the commencement and during the study was conducted on 28 adult patients attending a teaching dental hospital. The kappa values obtained for DMFT, CPI, LoA and VPI were 0.94, 0.70, 0.76 and 0.68, respectively. Duplicate examination of the participants was not possible; thus, intra-examiner reliability was not monitored.

The study protocol was reviewed by the Institutional Review Board (IRB) of the University of Hong Kong (IRB ref. no. UW 09–230), and ethical approval was granted before the implementation of this study. The reporting of this paper follows the STROBE statement.

### Data analysis

**Fig. 2:**
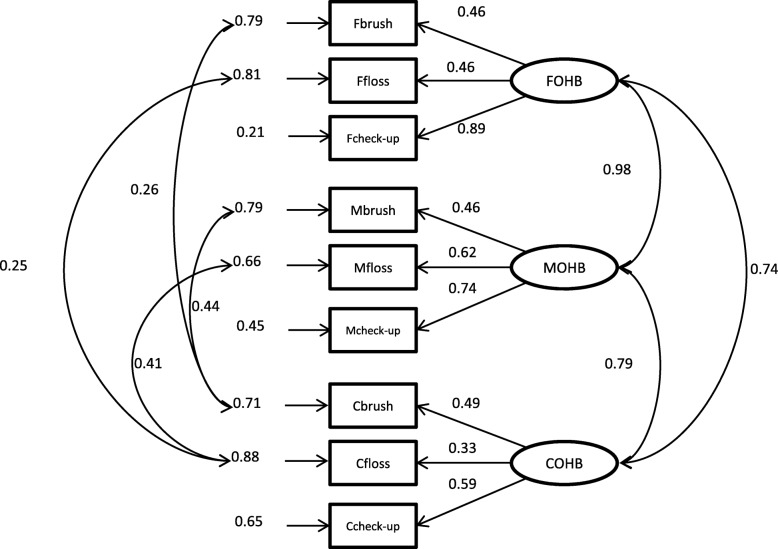
Path diagram of Family OHB model

**Fig. 3:**
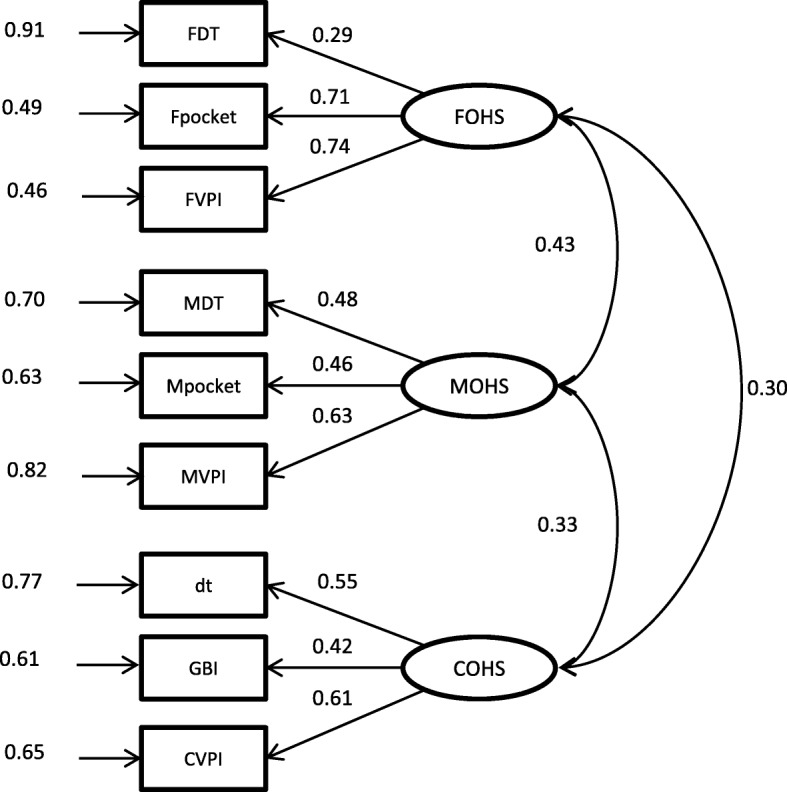
Path diagram of Family OHS model

The measurements of the latent variables in the hypothesized model of parental influences on children’s oral health practice and status (Fig. 1) are shown in Table 1. A moderate sample size of at least 400 would be needed for investigating the hypothesized model in this study [29, 30]. Before testing this hypothesized model, the correlation in the OHB among the parents and their children (Family OHB model, Fig. 2) and the correlation in the OHS among the parents and their children (Family OHS model, Fig. 3) were evaluated. The capital letters F, M, and C added before the abbreviation of the variable names refers to the data from fathers, mothers, and children, respectively. Additionally, POHKA stands for parents’ oral health knowledge and attitude towards children. Using LISREL 8.8, confirmatory factor analysis models were fitted to investigate the correlations of the latent variables among the family members. Then SEM was fitted to test whether the data behaved consistently with the hypothesized structural model. Maximum likelihood method for the parameter estimation was performed with the use of the polychoric matrix (which is recommended when the model includes both categorical and continuous observed variables). The standardized solution of the estimates was computed. A standardized parameter estimate for directional linkage (single arrow) shows the resulting change in a dependent variable from a standard deviation change in an independent variable. A standardized parameter estimate for non-directional linkage (double arrow) shows a correlation between variables involved [31]. In this study, comparative fit index (CFI) > 0.95, non-normed fit index (NNFI) > 0.95, and root mean square error of approximation (RMSEA) < 0.05 were regarded as a good fit. The Satorra and Bentler scaled chi-square test was used to adjust the model chi-square for non-normality [32]. The χ^2^/df < 2 was employed as an indicator that the data were well-fitted to the models. The R^2^ indicates the degree to which the observed variables are free from measurement error. The closer to 1 the value of R^2^ is, the better the observed variable indicating the corresponding latent variable is. The *t*-values greater than |1.96| indicate that the parameter estimates were significantly different from zero. Modification indexes > 10 were used as a reference to improve model specification when the model fit was poor.

**Table 1:** Measurement of latent variables

Latent variable	Observed variable	Abbreviation	
Socioeconomic status (SES)	Education level ^a^	edu	1 = Primary 2 = Secondary 3 = Post-secondary and Tertiary
	Family income per month ^a^	income	1 = <HKD10,000 2 = HKD10,000-HKD20,000 3= > HKD20,000
Oral health knowledge and attitude (OHKA)	Oral health knowledge score ^b^	know	Ranged from 0 to 12
	Oral health attitude score ^b^	atti	Ranged from 0 to 8
Parents’ oral health knowledge and attitude towards children (POHKA)	Knowledge to deal with child’s oral problem ^a^	knowledge	0 = Incorrect 1 = Correct
	Oral health attitude score ^b^	attitude	Ranged from 0 to 4
Parents’ oral health behaviors (OHB)	Tooth brushing frequency per day ^a^	brush	0 = Less than twice a day 1 = Twice a day or more
	Daily use of dental floss ^a^	floss	0 = No, 1 = Yes
	Regular dental visit ^a^	checkup	0 = Irregular, 1 = Regular
Children’s oral health behaviors (COHB)	Tooth brushing frequency per day ^a^	Cbrush	0 = Less than twice a day 1 = Twice a day or more
	Daily use of dental floss ^a^	Cfloss	0 = No, 1 = Yes
	Regular dental visit ^a^	Ccheckup	0 = Irregular, 1 = Regular
Parents’ oral health status (OHS)	Number of decayed teeth ^b^	DT	
	Presence of periodontal pocket ^a^	pocket	0 = Absence of periodontal pocket 1 = Presence
	Visual plaque index ^b^	VPI	Ranged from 0 to 100%
Children’s oral health status (COHS)	Number of decayed teeth ^b^	dt	
	Gingival bleeding index ^b^	GBI	Ranged from 0 to 100%
	Visual plaque index ^b^	CVPI	Ranged from 0 to 100%
Lifestyle	Daily smoker or not ^a^	smoke	0 = No smoking, 1 = Smoking

^a^Categorical variable

^b^Continuous variable

## Results

A total of 432 families agreed to participate in this study and 128 families declined our invitation. Among these families, 335 were from the FAMILY project and 97 were from the selected kindergartens. All children completed the dental examination, and 359 fathers and 419 mothers completed both of the questionnaires and the dental examination. The characteristics of the studied families are shown in Table 2.

**Table 2:** Characteristics of the studied families

	Fathers *n* = 359		Mothers *n* = 419		Children *n* = 432	
	n	%	n	%	n	%
Family income (HK$)^b^						
< 20,000	/	/	/	/	217	50.2
20,000–29,999	/	/	/	/	80	18.5
30,000 or above	/	/	/	/	135	13.3
Education level						
Primary	33	9.2	37	8.8	/	/
Secondary	99	27.6	125	29.8	/	/
Post-secondary and Tertiary	227	63.2	257	61.3	/	/
Oral health knowledge score ^a^ mean (SD)	8.1	(2.98)	8.8	(2.69)	/	/
Parents’ oral health knowledge towards children						
Incorrect	/	/	/	/	193	44.7
Correct	/	/	/	/	239	55.3
Oral health attitude score ^a^ mean(SD)	6.5	(1.26)	6.6	(1.23)	/	/
Parents’ oral health attitude towards children ^a^ mean(SD)	/	/	/	/	3.4	(0.80)
Tooth brushing						
< twice a day	123	34.3	75	17.9	114	26.4
≥ twice a day	236	65.7	344	82.1	318	73.6
Use of dental floss						
Yes	101	28.1	175	41.8	86	19.9
No	258	71.9	244	58.2	346	80.1
Regular dental checkup						
No	268	74.7	299	71.4	291	67.4
Yes	91	25.3	120	28.6	141	32.6
Daily smoker						
No	267	74.4	398	95.0	/	/
Yes	92	25.6	21	5.0	/	/
Presence of periodontal pocket(s)						
No	174	48.4	273	65.1	/	/
Yes	185	51.6	146	34.9	/	/
GBI ^a^ mean(SD)	/	/	/	/	0.23	(0.15)
DT/dt ^a^ mean(SD)	0.7	(1.20)	0.6	(1.24)	1.9	(3.18)
VPI ^a^ mean(SD)	0.50	(0.18)	0.48	(0.24)	0.41	(0.18)

^a^ instead of n and %, the cells show mean and standard deviation (SD) respectively

^b^Monthly household incomes were reported by 322 families, while those of the remaining 110 families were imputed based on their housing types [26]

Data from 346 families with complete information were used for the Family OHB model and Family OHS model. The initial Family OHB model did not fit well. The modification index suggested considering correlation among the measurement errors of some indicators. Because the same instrument was used to measure the same OHB within a family, it was reasonable to correlate the measurement error of the behavior among the family members. Four correlations between the measurement errors were added one-by-one according to the recommendation suggested by the modification index (Fig. 2). After modification, the model fitted well. NNFI was 0.988, CFI was 0.993, RMSEA was 0.039, and the chi-square value for the structural model was 30.3 with 20 degrees of freedom (χ^2^/df = 1.52, *P* = 0.07). In this model, all the indicators corresponded well with the latent variables. Strong positive correlations were obtained between the OHB of fathers and mothers (ϕ = 0.98, *P* < 0.001), mothers and children (ϕ = 0.79, *P* < 0.001), and fathers and children (ϕ = 0.74, *P* < 0.001).

For the Family OHS model, all the observed variables corresponded with the latent variables (Fig. 3). The goodness of fit indices RMSEA, NNFI, and CFI were 0.017, 0.991, and 0.994, respectively, all of which indicated a good fit. The chi-square value for the structural model was 26.49 with 24 degrees of freedom (χ^2^/df = 1.10, *P* = 0.33). The goodness-of-fit indices were satisfactory. The OHS between fathers and mothers was positively correlated (ϕ = 0.43, *P* < 0.001). Children’s OHS was also positively correlated with those of their mothers (ϕ = 0.33, *P* < 0.01) and fathers (ϕ = 0.30, *P* < 0.01).

**Fig. 4:**
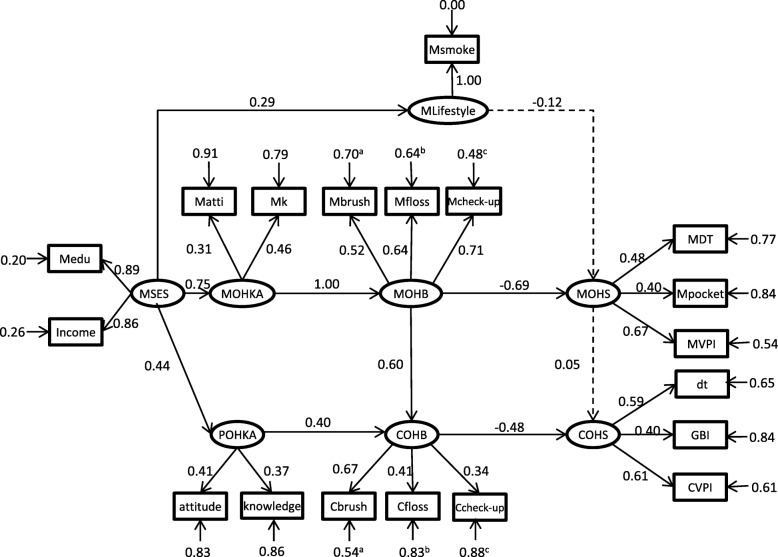
Path diagram of Mother-child OHS model

Because the Family OHB model (Fig. 2) and Family OHS model (Fig. 3) showed that OHB and OHS between father and mother were substantially correlated; only one parent’s data could be used to assess a child’s OHS. Because more mothers completed the questionnaire, the mother-child pair was used to explain the influence of the parental factors on their children’s oral health. Data from 419 mother-child pairs without missing information were used and fitted by the model. The path diagram of the Mother-child OHS model is shown in Fig. 4. Results showed that better socioeconomic status of mothers (MSES), reflected by higher education level and income, led to better oral health knowledge and attitude (MOHKA) (γ = 0.75, *P* < 0.001) and also towards their children (POHKA) (γ = 0.44, *P* < 0.01). POHKA (β = 0.40, *P* < 0.01) and mothers’ oral health behaviors (MOHB) (β = 0.60, *P* < 0.001) were positively associated with OHB of the children (COHB). Positive COHB (β = − 0.48, *P* < 0.01) in turn led to better oral health of children (COHS). However, mothers’ OHS (MOHS) did not show a significant relationship with COHS (β = 0.05, *P* = 0.70). These findings suggested that the children’s OHS was directly affected by their OHB and indirectly affected by their socioeconomic status, mother’s oral health knowledge and attitude, and OHB. The explained variance of children’s OHS was 26.3%. RMSEA was 0.023, NNFI was 0.990, and CFI was 0.991. The chi-square value for the structural model was 171.17 with 141 degrees of freedom (χ^2^/df = 1.241, *P* = 0.04). The goodness-of-fit indices indicated a good fit.

## Discussion

Results from this study show that OHB and OHS among the father, mother, and child in the same family were significantly correlated. The children’s OHS can be directly affected by their OHB, whereas their OHB can be directly affected by parental knowledge of and attitudes towards them and mothers’ OHB. The study population was children aged 5 to 7 and their parents in Hong Kong, with families recruited from various geographical districts. As discussed in a previous report, this study’s sample of children aged 5 to 7 and their parents can represent those in the Hong Kong population [26], the results from this study can be generalised in the local settings. However, the generalizability of the results in other dental service systems outside Hong Kong would require further testing. The limitation of this study is the use of SEM to analyse cross-sectional data. Since this is a cross-sectional study, the sequence of events and the changes by time cannot be identified [26]; other models could also be fitted and alternative ways to summarise the data are possible. Besides, duplicate examination of the participants was not possible; thus, intra-examiner reliability was not monitored.

In this study, a parental proxy questionnaire (from one parent, usually the mother) was used to collect the information of the children. Children, especially young children, usually cannot accurately report their behaviors because they may not have sufficient reading or expressive verbal ability. Some researchers have studied the agreement between a father and a mother when reporting information about their child. A meta-analysis of 60 studies with quantitative data that reported the emotional and behavioral problems of children and adolescents from both of their parents found that parental ratings were more correlated in behavioral information than in emotional information [33]. Later studies also found a strong agreement between fathers and mothers in providing both behavioral and emotional information of their children [34, 35]. Because the focus of this study was on the children’s OHB rather than emotional problems, the information from one parent was considered to be adequately reliable. Also, our findings show that mothers and children have stronger correlations in OHB and OHB than those between the fathers and children; mothers have more influences on their children than the fathers; thus, completion of the children’s questionnaire by the mothers could be justified.

OHB were significantly correlated among the father, mother, and child in the same family. In the Family OHB model, the correlations between errors of the observed variables were suggested by modification indices. Allowing correlations between measurement errors can improve the model fit. However, this practice may be debatable [36]. Correlating the measurement errors is justified when two indicators share something in common such as multiple measures of the same construct in a longitudinal study and different indicators using the same measure [37]. In this study, the same questions were used to collect information about OHB of fathers, mothers, and children. Therefore, it was reasonable to add the correlations between errors of the same behavior among fathers, mothers, and children. Based on the modification indices, appropriate correlations between errors were added one at a time. Then the model was reanalyzed, and one more correlation between errors was added until the modification indices suggested no further error covariance.

In this study, the correlations of OHB between the parents and children were lower than that between spouses but still high. The Hong Kong government provides primary school students with the School Dental Care Service program, which includes oral health education. Other organizations such as the Faculty of Dentistry at the University of Hong Kong and the Hong Kong Dental Association have carried out projects to improve preschool children’s oral health in recent years. Children aged 5 to 7 may have improved their OHB through these projects. So in this study, the correlations between parents and children were not as high as that between spouses. The correlations of OHB among family members suggest that when promoting good OHB, the family should be considered as a basic unit. Thus, the effectiveness and sustainability of oral health education can be enhanced.

The Family OHS model in this study showed that the OHS of fathers, mothers, and children were positively correlated. Furthermore, it can be seen that the correlation between a father and mother is higher than that between parents and child. Participation in the School Dental Care Service program and free dental care probably had improved the children’s oral health irrespective of their parents’ behaviors. The program would have weakened the correlation between the OHS of the parents and that of their child.

Parents can influence their children’s oral health, especially in the mother-child pair. The Family OHB model and Family OHS model showed that the OHB and OHS among family members were positively correlated. The correlation between mother and child is stronger than that between father and child. It is probably because a mother is usually the primary caregiver in the early stage of a child’s life. Therefore, mothers may play a more critical role in shaping their children’s oral health. The pathways from mothers’ socioeconomic statuses, through parental oral health knowledge and attitudes towards their children and children’s OHB to children’s OHS are consistent with the findings from Singapore preschool children [38]. These pathways showed that mothers with lower socioeconomic status had poorer oral health knowledge and attitudes towards their children. The mothers with better oral health knowledge and attitudes towards the children had children with better OHB and OHS. These findings suggest that more attention should be paid to children from lower socioeconomic backgrounds. Intervention such as proper oral health education of mothers may improve children’s OHB and OHS.

Mothers’ oral health practices contributed more than knowledge and attitudes to children’s oral health practices. This finding suggested that children aged 5 to 7 are likely to imitate their parents’ OHB. From this finding, it can be deduced that good oral health practices of a mother could benefit her children and herself. The finding suggests that oral health promotion for parents may improve their OHS and also their children’s OHS through changing their children’s oral health practices.

## Conclusions

The correlations of OHB and OHS between mothers and children were stronger than those between fathers and children. The children’s OHS can be directly affected by their OHB, whereas their OHB can be directly affected by parental knowledge of and attitudes towards them and parents’ OHB. Findings from this study support that family plays a significant role in determining an individual’s OHS. It was found that spouses’ OHB and OHS were highly correlated among parents and their children in this study sample. These findings imply that oral health promotion in Hong Kong should pay more attention to the whole family instead of individuals. In future oral health promotion activities, all family members should be involved to improve the effect on the promotion.

## Supplementary information

**Additional file 1.** Appendix 1 Questionnaires used in the study.

## Data Availability

The dataset analysed in the current study are available from the corresponding author on reasonable request.

## Abbreviations

CFI: Comparative fit index
CPI: Community periodontal
GBI: Gingival bleeding index
LOA: Loss of attachment
MOHB: Mothers’ oral health behaviors
MOHKA: Mothers’ oral health knowledge and attitude
MOHS: Mothers’ oral health status
MSES: Socioeconomic status of mothers
NNFI: Non-normed fit index
OHB: Oral health behaviors
OHB: Oral health behaviors
OHS: Oral health status
OHS: Oral health status
POHKA: Parents’ oral health knowledge and attitude towards children
RMSEA: Root mean square error of approximation
SEM: Structural equation model
VPI: Visible plaque index
