# Supplementary material for: Structural equation model for parental influence on children’s oral health practice and status
Source: BMC Oral Health. 2020 Feb 17;20:56. doi: 10.1186/s12903-020-1048-2 (PMC7027297; doi:10.1186/s12903-020-1048-2)
Supplement: Supplementary file 1 — Additional file 1. Appendix 1 Questionnaires used in the study. [file 12903_2020_1048_MOESM1_ESM.pdf]

Household # ☐☐☐☐☐☐☐
Identity # ☐

Adult Questionnaire

Name: ______________ Gender： ☐ M ☐ F Survey date: ______________

A. Your diet and oral health habits

1. How often do you brush your teeth every day?

☐ 1. Less than once / occasionally
☐ 2. Once
☐ 3. Twice
☐ 4. Three times or more

2. Does the toothpaste you have been using contain fluoride?

☐ 1. With fluoride
☐ 2. Without fluoride
☐ 3. I do not know whether the toothpaste contain fluoride or not
☐ 4. I do not use toothpaste

3. Do you use additional oral cleaning aids?

☐ 1.No ☐ 2. Yes (Multiple choices) ☐ 1. dental floss
☐ 2. mouth rinse
☐ 3. other place, please specify__

4. In the past 2 weeks, how many times do you intake the following snacks **on average every day**?

a)	Candy/Chocolate	☐ 1. < once	☐ 2. 1-2 times	☐ 3. 3 times or more
b)	Soft/sugary drinks	☐ 1. < once	☐ 2. 1-2 times	☐ 3. 3 times or more
c)	Cake/sugary snacks (biscuits)	☐ 1. < once	☐ 2. 1-2 times	☐ 3. 3 times or more

B. <u>Dental service utilisation</u>

5. Do you have dental scheme coverage?

☐ 1. No dental scheme
☐ 2. Scheme provided by employers
☐ 3. Self-purchased dental insurance/plan
☐ 4. Others, please specify______________

6. Do you visit dentist regularly?

☐ 1.Yes
☐ 2. No

7. Have you been to a dentist?

☐ 1. No (skip to Q.10)
☐ 2. yes (proceed to Q7a & 7b)

7a. The reasons for your last visit to the dentist were: (Multiple choices)

☐ 1. Go for check-up/ cleaning
☐ 2. Tooth decay
☐ 3. Toothache/abscess/other tooth discomfort
☐ 4. Tooth trauma
☐ 5. Go for treatment
☐ 6. Other reasons, please specify______________

7b. When was your last dental visit?

☐ 1. Less than 6 months
☐ 2. 7-12 months
☐ 3. 1-3 years
☐ 4. More than 3 years

C. Your oral health knowledge

8. What do you know about the factors leading to tooth decay? (Multiple choices)

☐ 1. Too much candies or sweet food
☐ 2. Bacteria/plaque
☐ 3. Lack of calcium
☐ 4. Improper toothbrushing
☐ 5. No regular dental check up
☐ 6. Other reason, please specify______________
☐ 7. Do not know

9. What do you know about the prevention of tooth decay? (Multiple choices)

☐ 1. Reduce candies and sweet food
☐ 2. Take calcium supplement
☐ 3. Proper toothbrushing
☐ 4. Use fluoride toothpaste
☐ 5. Seek regular dental check up
☐ 6. Other reason, please specify______________
☐ 7. Do not know

10. What do you know about factors leading to gum disease? (Multiple choices)

☐ 1. Bacteria/plaque
☐ 2. Poor nutrition/lack of vitamin
☐ 3. Improper toothbrushing
☐ 4. Traditional Chinese medical belief, such as "Hot air"
☐ 5. No regular dental check up
☐ 6. Other reason, please specify______________
☐ 7. Do not know

11. What do you know about prevention of gum disease? (Multiple choices)

☐ 1. Take vitamin / nutrient supplement
☐ 2. Proper toothbrushing
☐ 3. Drink herbal tea
☐ 4. Seek regular dental check up
☐ 5. Use medicated mouth rinse
☐ 6. Other reason, please specify______________
☐ 7. Do not know

D. <u>Your oral health status and attitude</u>

12. What do you think about your oral health status?

☐ 1. Very good
☐ 2. Good
☐ 3. Fair
☐ 4. Poor
☐ 5. Very Poor

13. Do you think that your oral health status have any effect on your life overall?

☐ 1. No effect
☐ 2. Little effect
☐ 3. Moderate effect
☐ 4. Great effect
☐ 5. Extremely great effect

14. Do you agree with the following statement?

	Agree	Disagree	Don't know
a. Just like ageing and death, loss of teeth is a natural process.	☐	☐	☐
b. State of teeth is decided at birth and is not related to self-care.	☐	☐	☐
c. Poor teeth are detrimental to one's appearance.	☐	☐	☐
d. State of my teeth is not important to me.	☐	☐	☐
e. Keeping natural teeth is not important.	☐	☐	☐
f. Dental problem can affect the whole body.	☐	☐	☐
g. False teeth will be less a bother than natural teeth.	☐	☐	☐
h. Regular visits to the dentist can prevent dental problems	☐	☐	☐

15. **In the past 12 months**, did you ever suffer from the following oral health problem?
(Multiple choices)

☐ 1. Bad breath
☐ 2. Difficulty in chewing
☐ 3. Dryness of mouth on eating
☐ 4. Abscess
☐ 5. Bleeding gums
☐ 6. Mobile teeth
☐ 7. Pain that disturbed sleep
☐ 8. Sensitivity to hot or cold
☐ 9. No experience of the above problems

Impact of oral health on life quality

Within past 12 months

	Never	Hardly ever	Occasionally	Fairy often	Very often
16. Have you ever had trouble pronouncing any words because of problems with your teeth, mouth or dentures?	○	○	○	○	○
17. Have you felt that there has been less flavour in your food because of problems with your teeth, mouth or dentures?	○	○	○	○	○
18. Have you had painful aching in your mouth?	○	○	○	○	○
19. Have you found it uncomfortable to eat any food because of problems with your teeth, mouth or dentures?	○	○	○	○	○
20. Have you seen self-conscious because of your teeth, mouth or dentures?	○	○	○	○	○
21. Have you felt tense because of problems with your teeth, mouth or dentures?	○	○	○	○	○
22. Has your diet been unsatisfactory because of problemswith them?	○	○	○	○	○
23. Have you had to interrupt meals because of problems with your teeth, mouth or dentures?	○	○	○	○	○
24. Have you found it difficult to relax because of problems with your teeth, mouth or dentures?	○	○	○	○	○
25. Have you been a bit embarrassed because of problems with your teeth, mouth or dentures?	○	○	○	○	○
26. Have you been a bit irritable with other people because of problems with your teeth, mouth or dentures?	○	○	○	○	○
27. Have you had difficulty doing your usual jobs because of problems with your teeth, mouth or dentures?	○	○	○	○	○
28. Have you felt that life in general was less satisfying because of problems with your teeth, mouth or dentures?	○	○	○	○	○
29. Have you been totally unable to function because of problems of your teeth, mouth or dentures?	○	○	○	○	○

E. **Personal information & general health status**

30. Your occupation:

☐ 1. Managers and administrators
☐ 2. Professionals
☐ 3. Associate professionals
☐ 4. Clerks
☐ 5. Service workers and shop sales workers
☐ 6. Market-oriented skilled agricultural and fishery workers
☐ 7. Craft and related workers
☐ 8. Plant and machine operators and assemblers
☐ 9. Elementary workers
☐ 10. Others, please specify: ____________________________

31. Your education level:

☐ 1. Primary or below ☐ 2. Junior high school
☐ 3. Senior high school ☐ 4. Tertiary education (Bachelor degree)
☐ 5. Tertiary education (master degree or above)

32. Have you ever smoked cigarettes?

☐ 1. No, skip to Q34 ☐ 2. Yes, proceed to Q33

33. Do you smoke now/before?

☐ 1. Never
☐ 2. Quitted, for how long: ____________________
☐ 3. Sometimes, less than 1 cigarette every day (i.e. < 7 per week)
☐ 4. Yes, at least one every day (i.e. at least 7 per week), please specify the number per day: ___

34. Do you drink now/before?

☐ 1. Never
☐ 2. < once per month
☐ 3. 1-3 times per month
☐ 4. 1-3 times per week
☐ 5. 4-6 times per week
☐ 6. Everyday
☐ 7. Quitted, for how long: ____________________

35. What is your usual level of consumption in a typical week?

	Can	Large bottle	Small bottle	Large beer mug	Small beer mug	Water glass	Wine glass	Spirit glass	Spirit shot glass	Small Chinese cup
**Beer**										
**Talbe wine**										
**Spirits**										
**Chinese wine**										
**Others**										

36. In the past month, how often have you consumed the following beverages?

	Less than once a month'	1-3 days per month	1-3 days per week	4-6 days per week	Every day
**Milk/Milk power**					
**Chinese herbal tea**					
**Pop/soft drinks**					
**Juice**					

37. Do you have the following systemic disease? (Multiple choices)

a) Diabetes mellitus ☐ 1. No ☐ 2. Yes
b) Hypertension ☐ 1. No ☐ 2. Yes
c) Hypercholesterolemia ☐ 1. No ☐ 2. Yes
d) Heart disease ☐ 1. No ☐ 2. Yes
e) Asthma ☐ 1. No ☐ 2. Yes
f) Other disease, please specify: _______________ ☐ 1. No ☐ 2. Yes

38. What do you think about your general health status?

☐ 1. Very good ☐ 2. Good ☐ 3. Fair
☐ 4. Poor ☐ 5. Very Poor

39. Your date of birth: ____ Year ____ Month

Height: _____ cm Weight: _____ kg

~~ Finished ~~

Household # ☐☐☐☐☐☐☐
Identity # ☐

Child Questionnaire

Name of child: ________ Gender: ☐ M ☐ F Age: ______________
Place of birth: ☐ Hong Kong ☐ China ☐ Others place, please specify_____
Surrvey date:____________-

A. Your child's diet and oral health habits

1. How often does your child brush the teeth every day?

☐ 1. Less than once / occasionally
☐ 2. Once
☐ 3. Twice
☐ 4. Three times or more

2. How old was your child when he/she started brushing the teeth?

☐1. 6-12 months ☐2. 13-18 months ☐3. 19-24 months ☐4. > 24 months

3. Does the toothpaste your child has been using contain fluoride?

☐ 1. With fluoride
☐ 2. Without fluoride
☐ 3. I do not know whether the toothpaste contain fluoride or not
☐ 4. I do not use toothpaste

4. Does your child use additional oral cleaning aids?

☐ 1.No ☐ 2. Yes (Multiple choices)
☐ 1. dental floss
☐ 2. mouth rinse
☐ 3. other place, please specify______

5. In the past 2 weeks, how many times does your child intake the following snacks **on average every day**?

a)	Candy/Chocolate	☐ 1. < once	☐ 2. 1-2 times	☐ 3. 3 times or more
b)	Soft/sugary drinks	☐ 1. < once	☐ 2. 1-2 times	☐ 3. 3 times or more
c)	Cake/sugary snacks (biscuits)	☐ 1. < once	☐ 2. 1-2 times	☐ 3. 3 times or more

B. Dental service utilisation

6. Does your child have dental scheme coverage?

☐ 1. No dental scheme
☐ 2. Scheme provided by employers
☐ 3. Self-purchased dental insurance/plan
☐ 4. School Dental Care Service
☐ 5. Others, please specify_______________

7. Does your child visit dentist regularly?

☐ 1.Yes
☐ 2. No

8. Has your child been to a dentist?

☐ 1. No (skip to Q.9)
☐ 2. yes (proceed to Q8a & 8b)

8a. The reasons for last visit to the dentist were: (Multiple choices)

☐ 1. Go for check-up/ cleaning
☐ 2. Tooth decay
☐ 3. Toothache/abscess/other tooth discomfort
☐ 4. Tooth trauma
☐ 5. Go for treatment
☐ 6. Other reasons, please specify_______________

8b. When was the last dental visit?

☐ 1. Less than 6 months
☐ 2. 7-12 months
☐ 3. 1-3 years
☐ 4. More than 3 years

C. <u>Your oral health attitude towards your child</u>

9. What do you think about your child’s oral health status?

☐ 1. Very good
☐ 2. Good
☐ 3. Fair
☐ 4. Poor
☐ 5. Very Poor

10. Do you think that your child’s oral health status have any effect on his/her life overall?

☐ 1. No effect
☐ 2. Little effect
☐ 3. Moderate effect
☐ 4. Great effect
☐ 5. Extremely great effect

11. Do you agree with the following statement?

	Agree	Disagree	Don’t know
a. Keeping primary teeth of your child health is as important as their permanent teeth.	☐	☐	☐
b. The tooth decay of primary teeth of child can be ignored because the primary teeth will be exfoliated later.	☐	☐	☐
c. Dental problem of primary teeth of your child can affect his/her whole body.	☐	☐	☐
d. Regular visits to the dentist can keep your child’s primary teeth.	☐	☐	☐

12. If your child had decay in a baby tooth what treatment would you want?

☐ 1. Leave it alone, because it will be replaced by permanent tooth later
☐ 2. Exact it ☐ 3. Fill it
☐ 4. Others, please specify__________ ☐ 5. Do not know

D. Impact of oral health on life quality

Problems with the teeth, mouth or jaws and their treatment can affect the well-being and everyday lives of children and their families. For each of the following questions please circle the number next to the response that best describes your child's experiences or your own. Consider the child's entire life from birth until now when answering each question. If a question does not apply, check "Never"

	**Never**	**Hardly ever**	**Occas-ionally**	**Often**	**Very often**	**Don't know**
13. How often has your child had pain in the teeth, mouth or jaws?						
How often has your child......because of dental problems or dental treatments?						
14. had difficulty drinking hot or cold beverages						
15. had difficulty eating some foods						
16. had difficulty pronouncing any words						
17. missed preschool, daycare or school						
18. had trouble sleeping						
19. been irritable or frustrated						
20. avoided smiling or laughing when around other children						
21. avoided talking with other children						
How often have you or another family member......because of your child's dental problems or dental treatments?						
22. been upset						
23. felt guilty						
How often....						
24. have you or another family member taken time off from work .....because of your child's dental problems or dental treatments						
25. has your child had dental problems or dental treatments that had a financial impact on your family?						

E. Medical history & general health status of your child

26. In general, how would you rate your child's health?

☐ 1. Very good ☐ 2. Good ☐ 3. Fair
☐ 4. Poor ☐ 5. Very Poor

27. Does your child have long-term medication/medical services?

☐ 1. No ☐ 2. Yes, please specify________________-

28. In the past four weeks, how much bodily pain or discomfort has your child had?

☐ 1. Never ☐ 2. Very slight ☐ 3. Slight
☐ 4. Fair ☐ 5. Serious 6. Very serious

29. In the past four weeks, how often has your child had bodily pain or discomfort?

☐ 1. Never ☐ 2. Very slight ☐ 3. Slight
☐ 4. Fair ☐ 5. Serious 6. Very serious

30. In the past four weeks, how many times does your child go to Chinese/Western clinics?
Please specify____________

F. <u>**Daily physical activity of your child**</u>

In the past four weeks

31. Has your child been limited in any of the following activities **due to health problems**?	**Without limitation**	**Little limitation**	**Some limitation**	**A lot of limitation**
Doing things that take a lot of energy, such as playing soccer or running				
Doing things that take some energy such as riding a bike or skating				
Ability (physically) to get around the neighborhood, playground, or school				
Walking one block or climbing one flight of stairs				
Bending, lifting/stooping; taking care of him/herself?				
32. Has your child's school work or activities with friends been limited in any of the following ways **due to** emotional **difficulties or problems with his/her behavior**?				
Limited in the kind of schoolwork or activities with friends he/she could do				
Limited in the amount of time he/she could spend on schoolwork or activities with friends				
Limited in performing schoolwork or activities with friends?				
Has your child's school work or activities with friends been limited in any of the following ways **due to problems with his/her physical health**?				
Limited in the kind of schoolwork or activities with friends he/she could do				
Limited in the amount of time he/she could spend on schoolwork or activities with friends?				

~ Finished ~~
